# *Trypanosoma cruzi* Response to Sterol Biosynthesis Inhibitors: Morphophysiological Alterations Leading to Cell Death

**DOI:** 10.1371/journal.pone.0055497

**Published:** 2013-01-31

**Authors:** Rafael Luis Kessler, Maurilio José Soares, Christian Macagnan Probst, Marco Aurélio Krieger

**Affiliations:** Instituto Carlos Chagas, FIOCRUZ, Curitiba, PR, Brazil; Albert Einstein Institute for Research and Education, Brazil

## Abstract

The protozoan parasite *Trypanosoma cruzi* displays similarities to fungi in terms of its sterol lipid biosynthesis, as ergosterol and other 24-alkylated sterols are its principal endogenous sterols. The sterol pathway is thus a potential drug target for the treatment of Chagas disease. We describe here a comparative study of the growth inhibition, ultrastructural and physiological changes leading to the death of *T. cruzi* cells following treatment with the sterol biosynthesis inhibitors (SBIs) ketoconazole and lovastatin. We first calculated the drug concentration inhibiting epimastigote growth by 50% (EC_50_/72 h) or killing all cells within 24 hours (EC_100_/24 h). Incubation with inhibitors at the EC_50_/72 h resulted in interesting morphological changes: intense proliferation of the inner mitochondrial membrane, which was corroborated by flow cytometry and confocal microscopy of the parasites stained with rhodamine 123, and strong swelling of the reservosomes, which was confirmed by acridine orange staining. These changes to the mitochondria and reservosomes may reflect the involvement of these organelles in ergosterol biosynthesis or the progressive autophagic process culminating in cell lysis after 6 to 7 days of treatment with SBIs at the EC_50_/72 h. By contrast, treatment with SBIs at the EC_100_/24 h resulted in rapid cell death with a necrotic phenotype: time-dependent cytosolic calcium overload, mitochondrial depolarization and reservosome membrane permeabilization (RMP), culminating in cell lysis after a few hours of drug exposure. We provide the first demonstration that RMP constitutes the “point of no return” in the cell death cascade, and propose a model for the necrotic cell death of *T. cruzi*. Thus, SBIs trigger cell death by different mechanisms, depending on the dose used, in *T. cruzi*. These findings shed new light on ergosterol biosynthesis and the mechanisms of programmed cell death in this ancient protozoan parasite.

## Introduction

Chagas disease, or American trypanosomiasis, is an endemic zoonosis in South and Central America caused by the protozoan parasite *Trypanosoma cruzi* [1], [2]. This disease remains a major public health problem and there is still no effective treatment or vaccine against *T. cruzi* [3], [4], [5]. New methods for the specific treatment of Chagas disease are thus urgently required. Several new drugs are of potential interest in this context [3], [6], including sterol biosynthesis inhibitors (SBIs) [7].

Trypanosomatids resemble fungi in terms of their endogenous biosynthesis and cellular composition of sterols [8]. Unlike mammals, which synthesize cholesterol, epimastigote forms of *T. cruzi* produce mostly ergosterol [9]. *T. cruzi* contains significant amounts of cholesterol of exogenous origin [9], mostly in amastigotes [10], but it remains highly susceptible to sterol biosynthesis inhibitors, demonstrating a need for specific sterols not synthesized by the host [11]. The sterol biosynthesis pathway is therefore considered one of the most attractive targets for the specific treatment of Chagas disease [7], and several enzymes from this pathway have been studied as possible treatment targets [11].

These potential target enzymes include the cytochrome P-450-dependent enzyme sterol 14-alpha-demethylase (C14-DMT), which is responsible for the demethylation of the C-14 in steroid rings [12]. This enzyme can be inhibited with azoles, a family of drugs initially developed as antifungal agents [13]. Azoles have been tested against *T. cruzi* for more than 20 years [12], and it has been shown that ketoconazole inhibits the growth of *T. cruzi in vitro* [14], [15], [16], by blocking the *de novo* biosynthesis of endogenous sterols [16], with inhibitory concentrations for amastigotes that are not toxic to host cells [12], [14]. However, studies in a murine model [17], [18] and in humans [18] have shown that ketoconazole is not effective at the chronic stage of the disease (reviewed by [12]). Several new azoles have recently been tested against *T. cruzi* [19], [20], [21], [22], [23], [24], with various degrees of success. Promising results were obtained with new triazole derivatives, including posaconazole, which eliminated the parasite in experimental models of acute and chronic Chagas disease [22], [24], [25], including benznidazole-resistant strains [24], . Posaconazole is currently undergoing initial trials in humans [7].

Another potentially interesting enzyme, acting on the initial steps of the sterol pathway, is 3-hydroxy-3-methylglutaryl CoA reductase (HMGR), which is inhibited by statins, a class of drugs studied for decades as a means of reducing plasma cholesterol levels and preventing coronary heart disease in humans by inhibiting the mevalonate pathway [27]. As the initial steps of sterol synthesis are similar in all eukaryotes, statins have also been tested against *T. cruzi* [28], [29], [30], [31]. Studies with lovastatin (mevenolin) have shown cell growth inhibition and cell lysis in cultures of *T. cruzi* epimastigotes, but very poor therapeutic activity *in vitro* against amastigote forms [29].

Given the potential importance of the sterol biosynthesis pathway as a major drug target for the treatment of Chagas disease, we analyzed the biological response of epimastigote forms of *T. cruzi* to classical SBIs, at both the cellular and molecular levels, as a first step toward a more extensive characterization of the *T. cruzi* response encompassing amastigote forms and other SBIs of greater theraupetic potential. We present here the results of a cellular analysis, including a comparative study of the growth inhibition, ultrastructural modifications and physiological changes leading to the death of *T. cruzi* epimastigotes in response to ketoconazole and lovastatin, as a function of drug concentration and exposure time. The molecular characterization, involving large-scale gene expression analysis, will be presented elsewhere.

In the presence of SBI concentrations capable of inhibiting growth in culture by 50% (EC_50_/72 h), the most affected organelles were mitochondria and reservosomes, leading to cell lysis only after six to seven days of exposure, with the presence of autophagic vacuoles and myelin figures. However, at higher doses capable of killing all parasites in less than 24 hours (EC_100_/24 h), the cells died by necrosis, with cell swelling and vacuolization, cytosolic calcium overload, mitochondrial depolarization, reservosome membrane permeabilization (point of no return) and time-dependent cell lysis with no classical markers of apoptosis (phosphatidylserine exposure and internucleosomal DNA degradation). These findings greatly increase our understanding of ergosterol biosynthesis and the mechanisms of programmed cell death in this ancient protozoan parasite.

## Materials and Methods

### Parasite

Epimastigote forms of *Trypanosoma cruzi* strain Dm28c were maintained in culture, at 28°C, without shaking, by weekly transfer to fresh liver infusion tryptose (LIT) medium [32] supplemented with 10% fetal bovine serum. Three-day-old cultured forms (in mid-exponential growth phase) were used for all experiments.

### Sterol biosynthesis inhibitors (SBIs)

Ketoconazole (an inhibitor of sterol 14-alpha-demethylase, C14-DMT) and lovastatin (mevenolin, an inhibitor of 3-hydroxy-3-methylglutaryl-CoA reductase, HMGR) were obtained from Sigma (Sigma, St. Louis, Co, USA). The compounds were dissolved in 100% DMSO (dimethyl sulfoxide) to obtain 50 mM stock solutions.

### Antiproliferative activity of SBIs *in vitro*

*T. cruzi* epimastigote cultures were set up with an initial cell density of 2×10^6^ cells/ml. Inhibitors were added the next day, when the cell density had reached ∼5×10^6^ cells/ml. Cell density was then analyzed daily, by direct counting in a hemocytometer (Neubauer chamber), for five days and cell viability was assessed by analyzing morphology and motility. The experiments were performed in triplicate and growth curves of cultures without drugs or with DMSO only were used as controls. The final concentration of DMSO in the cultures never exceeded 0.3% and had no effect on parasite growth (data not shown).

Growth inhibition was quantified by defining a percentage growth factor (%GF) based on a comparison of treated and untreated cultures [33], and the percentage growth inhibition (%GI) was estimated as %GI = 100 - %GF. The effective concentration (EC) of the drug required to reduce parasite proliferation by 50% was calculated by nonlinear regression analysis of %GI against drug concentration, with GraphPad Prism software. We used data from the fourth day of culture, corresponding to three days (72 hours) of inhibitory treatment (EC_50_/72 h).

We also determined the minimal concentration of drugs capable of killing all the cells in the culture within 24 hours of exposure (EC_100_/24 h). The parasites were considered dead when spheroid-shaped, static [34], [35], and unable to resume growth when transferred to drug-free medium (recovery experiments, see below). Unlike the EC_50_/72 h, this concentration was obtained empirically, by testing several high concentrations of the drugs.

### Recovery experiments

Cultured epimastigotes, at a cell density of 4–6×10^6^ cells/ml, were exposed to high doses of SBIs (90, 100, 110 and 120 µM) for short periods (15 minutes to 4 hours). About 5×10^6^ cells were then collected by centrifugation at 2000×*g* for 5 min, washed three times with sterile phosphate-buffered saline (PBS) and transferred to fresh, drug-free LIT medium at a density of 2×10^6^ cells/ml. Growth recovery was then monitored by determining cell density daily in a hemocytometer. Relative growth was obtained by determining the ratio of the cell density in drug-stressed cultures to that in control cultures, after three days of drug contact. The EC_100_/24 h was considered to be the minimal dose inhibiting subsequent growth of the culture after a short period of exposure (less than 4 hours), indicative of the activation of programmed cell death pathways.

### Transmission electron microscopy (TEM)

Parasites were collected by centrifugation at 7000×*g*, washed twice with PBS and fixed by incubation for 24 hours at room temperature with 2.5% glutaraldehyde in 0.1 M phosphate buffer (pH 7.2). Cells were washed twice in 0.1 M phosphate buffer, then post-fixed by incubation for one hour at room temperature in a solution containing 1% osmium tetroxide, 0.8% potassium ferricyanide, 5 mM calcium chloride, 0.1 M cacodylate buffer pH 7.2. The parasites were then dehydrated in increasing concentrations of acetone and embedded overnight in a 1∶1 mixture of 100% acetone and PolyBed 812 resin (Polysciences, Warrington, FL, USA). The samples were then embedded by incubation in pure PolyBed 812 resin for 4 to 6 hours and polymerization for 48 hours at 60°C. Ultrathin sections were obtained with a Leica EM UC6 ultramicrotome and contrast-stained by incubation for 40 min in 5% uranyl acetate and 2 min in lead citrate. The samples were analyzed in a Jeol JEM-2100 transmission electron microscope at the *Laboratório Central de Microscopia Eletrônica* (LCME, UFSC, Santa Catarina, BR) and the images were adjusted to improve the contrast with Adobe Photoshop CS2 software.

### Light microscopy (stained smears)

For the observation of morphological changes in response to the SBIs, we collected 1×10^6^ cells by centrifugation at 2000×*g* for 5 min. The cells were washed once with PBS, fixed by incubation for 30 minutes with 4% paraformaldehyde and washed with PBS. We then allowed 5×10^5^ cells in 10 µl to dry onto microscopic slides and stained these cells with “Panótico Rápido” (Laborclin, Pinhais, PR, Brazil). The slides were mounted in Permount® (Fisher Scientific) and examined with a Nikon E600 microscope. Images were acquired with the Image Pro program (Media Cybernetics, Bethesda, MD, USA) and processed with Adobe Photosphop CS2 software to improve contrast.

### Flow cytometry

Flow cytometry experiments were performed in a FACSCalibur machine (Becton-Dickinson, San Jose, CA, USA). In total, 20,000 events were acquired in the regions previously identified as corresponding to *T. cruzi* epimastigotes. The data were then analyzed with FlowJo software (Treestar software). All experiments were performed at least in triplicate.

Acridine orange (AO) was used as a fluorescent marker of reservosomes, as previously described [36], [37]. At least 5×10^5^ cells were collected by centrifugation at 2000×*g* for 5 minutes and washed with 1 ml PBS. The parasites were resuspended in 5 µg/ml AO in PBS and incubated for 15 min at 28°C. The cells were washed three times in PBS and immediately quantified. For data analysis, we considered only viable cells gated on the basis of forward (FSC) and side (SSC) scatter. The level of acidic vesicle (reservosome) staining, with respect to that for untreated parasites, was established by determining the fold-change (ratio: treated/non treated cells) of the geometric mean ([(X_1_).(X_2_)…(X_N_)]^1/N^, where X is the fluorescence intensity of each event and N is the total number of events) FL3 (670 nm band-pass filter) signal intensity.

As recently shown for necrotic cell death in *Dictyostelium* [38], AO can also be used to monitor the permeabilization of acidic vesicles. We therefore also used this stain to estimate the degree of reservosome membrane permeabilization (RMP). Parasites were collected by centrifugation and stained for 10 min with 1 µg/ml acridine orange. They were then immediately quantified, without washing, with the FL1 detector (530/30 nm band-pass filter). Data were analyzed for viable cells gated by FSCxSSC scatter, and cells with a high FL1-H signal intensity were considered to have ruptured reservosomes.

We analyzed mitochondrial membrane potential, by washing the parasites as described above and incubating them for 15 min at 28°C with 10 µg/ml rhodamine 123 (R123). The cells were washed three times with 1 ml PBS and immediately quantified by flow cytometry. Data analysis were analyzed for viable cells gated on the basis of FSCxSSC scatter. Relative mitochondrial membrane potential was determined by considering the fold-change in the geometric mean of FL1-H signal intensity.

For cell viability analysis, washed parasites were resuspended in 5 µg/ml propidium iodide (PI) in PBS and incubated for 15 min at 28°C. The cells were then immediately quantified, without washing. Cells positively stained in FL2-H (585/42 nm band-pass filter) were considered to be dead.

For the determination of intracellular calcium concentration, 5×10^5^ cells were collected by centrifugation, resuspended in 1 µM Fuo-4-AM (Invitrogen, Carlsbad, CA, USA) in PBS, with or without 1 mM EGTA, and incubated for 15 min at 28°C. The cells were washed twice with PBS (or 1 mM EGTA in PBS) and immediately quantified. Relative intracellular Fluo-4-AM fluorescence was obtained by determining the fold-change of the geometric mean of FL1-H signal intensity.

Possible exposure of the phospholipid phosphatidylserine at the cell membrane was analyzed with the PharMingen Annexin V-FITC Apoptosis Detection Kit (BD, Franklin Lakes, NJ, USA), according to the manufacturer's instructions. The parasites were incubated for 30 min at 28°C with annexin-V-FITC (AV) in annexin-V binding buffer (10 mM Hepes/NaOH pH 7.4, 140 mM NaCl, 2.5 mM CaCl_2_), and then for 15 min at 28°C with the vital dye PI. Unstained cells and cells stained independently with each dye were used to establish signal compensation from the detectors.

### DNA fragmentation analysis

Epimastigotes were exposed to the EC_100_/24 h of SBIs for various periods of time. We then collected 6×10^7^ cells by centrifugation and washed then with PBS. Total DNA was isolated as previously described [39] and quantified with a NanoDrop spectrophotometer. Purified DNA (5 µg) was separated by electrophoresis in a 1.5% agarose gel and stained with ethidium bromide. The DNA bands were visualized under UV light.

DNA fragmentation was also analyzed by *in situ* TUNEL (terminal deoxynucleotidyl transferase dUTP nick end labeling), with the Click-iT TUNEL AlexaFluor Imaging Assay (Invitrogen), used according to the manufacturer's instructions.

### Fluorescence microscopy

For the visualization of live parasites stained with AO or R123, 5×10^5^ cells (in 10 µl) were allowed to adhere to glass microscope slides, covered with a coverslip and immediately observed with either a Leica SP5 confocal scanning microscope or a Nikon E600 epifluorescence microscope. The images were processed with Adobe Photoshop CS2 software to improve contrast.

The acidotrophic fluorescent dye LysoTracker Red DND-99 (Invitrogen) was used to monitor the presence of acidic compartments in fixed cells. About 1×10^7^ cells were recovered by centrifugation at 2000×*g* for 5 min and washed with PBS. The cells were incubated for 30 min at 28°C in PBS containing 0.5 µM LysoTracker Red. The staining procedure was terminated by fixing the cells by incubation with 4% paraformaldehyde for one hour. The parasites were washed with PBS and placed in Teflon-delimited wells on 0.1% poly-L-lysine-coated slides, to which they were allowed to adhere for 30 minutes. The slides were then washed by immersion in PBS to remove excess non adherent parasites and the DNA was labeled by incubation with 20 µl of a 2 µg/µl solution of Hoechst 33342 (Invitrogen). The slides were washed five times, by immersion in PBS, and processed by adding 10 µl of *n*-propyl gallate (anti-fading solution) and sealing using a coverslip and sealer (nail enamel). The samples were observed under a Leica SP5 confocal scanning microscope and the images were processed with Adobe Photoshop software to improve contrast.

For the immunostaining of reservosomes, parasites were centrifuged for 5 min at 2000×*g*, washed with PBS and fixed by incubation for 30 min with 4% paraformaldehyde in PBS. The cells were washed in PBS and allowed to adhere to poly-L-lysine-coated slides for 15 min. They were then washed with PBS and permeabilized by incubation with 0.1% Triton ×100 in PBS for 2 minutes. The cells were washed three times and blocked by incubation overnight at 4°C with 1% bovine serum albumin (BSA) in PBS. They were then incubated for 60 minutes at 37°C with the anti-TcRBP40 [40] primary antibody diluted 1∶100 in 1% BSA/PBS. The slides were washed three times with PBS, incubated for 60 min at ambient temperature with a secondary antibody (goat anti-mouse AlexaFluor 488) diluted 1∶400 in 1% BSA/PBS and washed three times with PBS. Finally, the slides were processed by incubation with Hoechst 33342 for DNA staining and anti-fading solution was added, as described above.

## Results

### Antiproliferative and trypanocidal effect of SBIs

**Figure 1:**
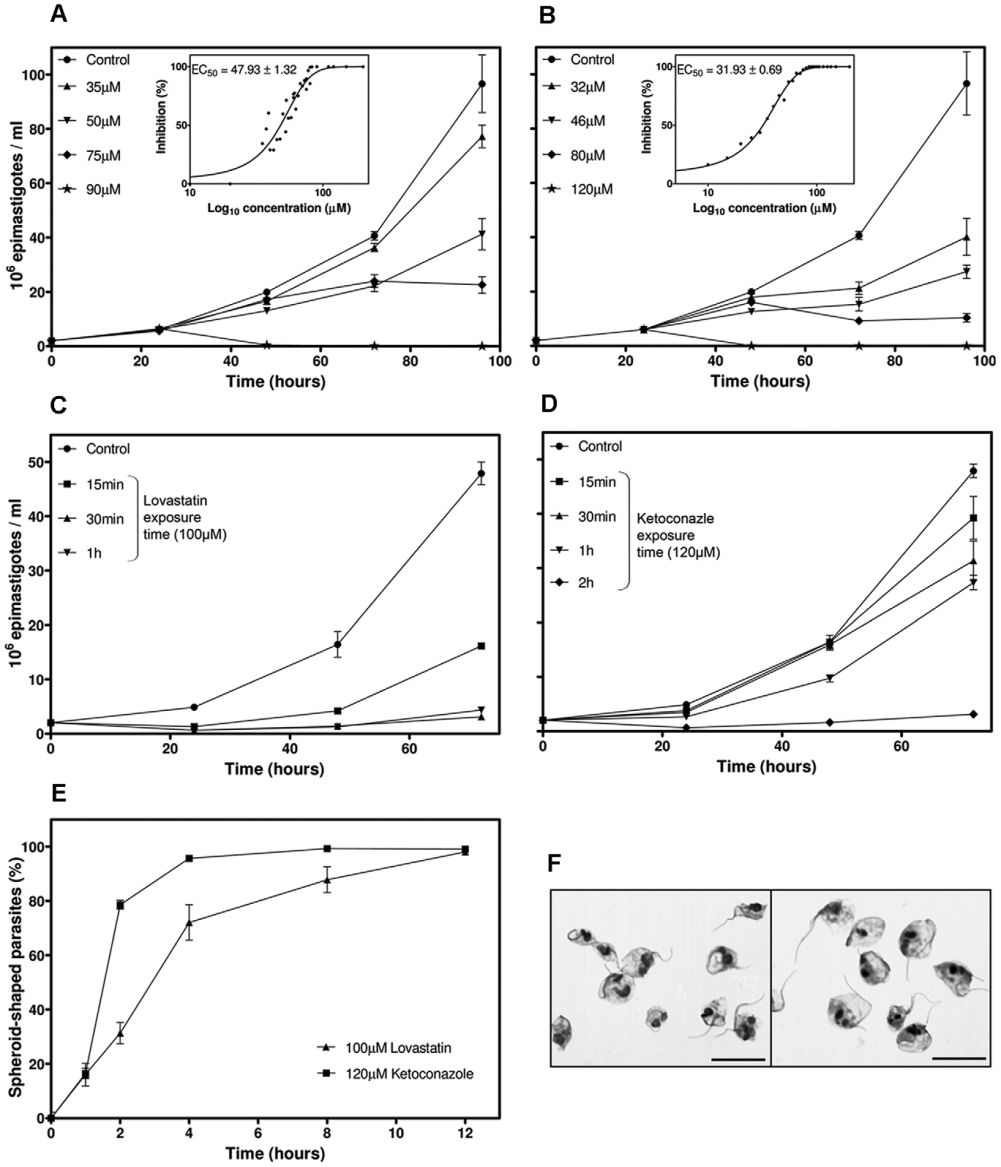
Antiproliferative and trypanocidal effects of SBIs in *T. cruzi*. (A and B) Growth curves of cultured epimastigotes exposed to various concentrations of lovastatin (A) or ketoconazole (B). The dose-response curve and respective EC_50_/72 h values are shown in the box. (C and D) Recovery experiments: epimastigote cultures were exposed to 100 µM lovastatin (C) or 120 µM ketoconazole (D). The drug was then removed by successive washes, after short periods of time (specified in the graph). The subsequent growth of the parasites was followed for three days, by counting, in a Neubauer chamber. (E) Percentage of dead cells (spheroid) as a function of time exposed to 120 µM ketoconazole or 100 µM lovastatin. (F) Stained smears of parasites exposed to SBIs at the EC_100_/24 h for 12 hours, showing the spheroid shape of the cells; the scale bars indicate 10 µm. For all graphs, each experimental point corresponds to the mean and standard deviation for cell density obtained by direct counting in a Neubauer chamber.

The incubation of *T. cruzi* epimastigotes with lovastatin resulted in concentration-dependent growth inhibition for concentrations of 20 µM to 80 µM (Figure 1A). The EC_50_/72 h was estimated at 48±1.32 µM from the drug response curve (Figure 1A, in box). A concentration-dependent growth inhibition effect was also observed for ketoconazole, for concentrations of 1 µM to 80 µM, with an estimated EC_50_/72 h of 32±0.69 µM (Figure 1B).

The minimal concentration of the drug capable of killing all cultured epimastigotes within 24 hours of exposure was also determined. Microscopic analysis of parasites incubated with high concentrations of lovastatin (90 to 110 µM) or ketoconazole (100 to 130 µM) showed that 100 µM of lovastatin and 120 µM of ketoconazole induced rapid and progressive cell death (Figure 1E). Dead parasites were defined as spheroid-shaped and static cells [35]. Exposure to SBIs for 12 hours resulted in the death of almost all the cells (Figure 1F). Cell death occurred more rapidly for ketoconazole than for lovastatin: ∼80% and ∼31% of the cells, respectively, were dead after exposure to the drug for two hours (Figure 1E).

Parasites treated for short periods of time with 100 µM lovastatin or 120 µM ketoconazole lost the ability to resume growth after the cessation of the drug stress, in a time-dependent manner. When transferred to drug-free medium, parasites incubated for 15 minutes with 100 µM lovastatin grew about 50% less than the control cells, whereas growth could not be re-established after 30 minutes of exposure (Figure 1C). The activation of cell death was slower after incubation with 120 µM ketoconazole, but one hour of exposure was sufficient to decrease the subsequent recovery rate to about 57% and two hours of drug contact was sufficient to prevent subsequent growth altogether (Figure 1D). At concentrations of lovastatin below 100 µM or of ketoconazole below 120 µM, there was no activation of a no-return cell death mechanism (data not shown). Based on these results, the EC_100_/24 h was set at 100 µM for lovastatin and 120 µM for ketoconazole.

### Morphophysiological changes and autophagy after treatment with SBIs at the EC_50_/72 h

Light microscopy analysis of stained smears showed that the exposure of *T. cruzi* epimastigotes to the EC_50_/72 h of lovastatin or ketoconazole induced a gradual swelling of the cells after four days of exposure, resulting in cell lysis after six to seven days (Figure S1). We also observed parasites with aberrant cell division processes containing two nuclei and/or kinetoplasts (Figure S1).

**Figure 2:**
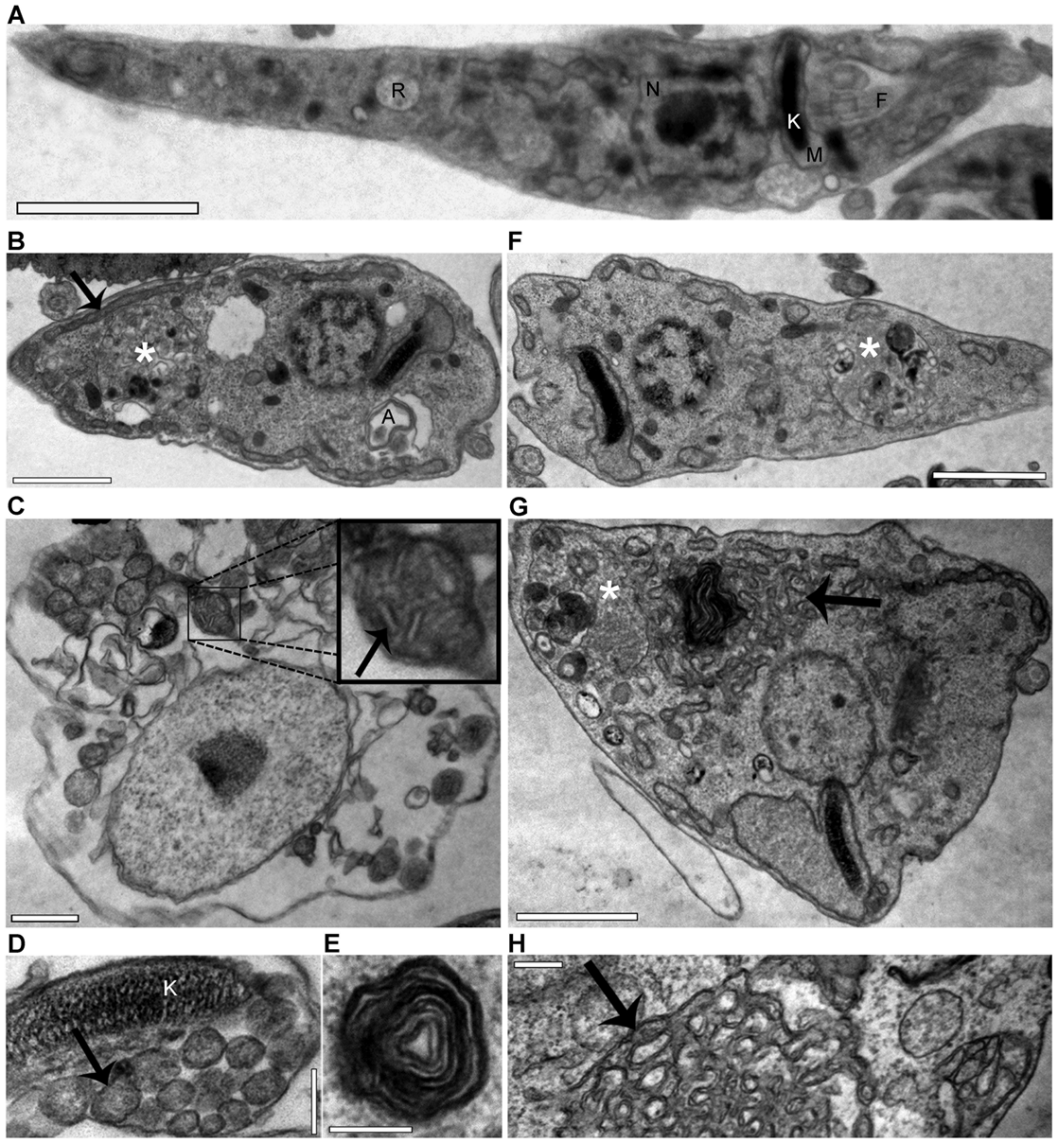
TEM of *T. cruzi* epimastigotes treated with SBIs at the EC_50_/72 h. (A) Control epimastigotes, providing a general view of parasite ultrastructure, indicating the nucleus (N), kinetoplast (K), mitochondria (M), flagellum (F) and reservosome (R). (B to E) Exposure to 50 µM lovastatin for 72 hours (B) or 120 hours (C to E). (F to H) Exposure to 32 µM ketoconazole for 72 hours (F) or 120 hours (G and H). For all images, the white asterisks (*) indicate the swollen reservosomes and the black arrows (→) indicate aberrant mitochondrial branching. The abnormal mitochondrial pattern is highlighted in (C) (in box). A myelin figure, typical of autophagic cells, is highlighted in (E). A: Autophagosome. Bars: (A), 2 µm; (B), (F), (G), 1 µm; (C), 0.5 µm; (D), (E), (H), 0.2 µm.

TEM analysis showed drastic ultrastructural changes in response to the EC_50_/72 h of both SBIs. Myelin figures (Figure 2G, in detail in Figure 2E) and autophagosome-like vacuoles (Figure 2B) were commonly observed, suggesting that the drugs caused autophagic cell death, as already observed for some SBIs in *T. cruzi* and *Leishmania* [11]. One remarkable change was the significant swelling of the reservosomes observed in response to both drugs. This organelle was extremely hypertrophied, and was at least twice as large as in control cells (white asterisks in Figure 2B, 2F and 2G).

**Figure 3:**
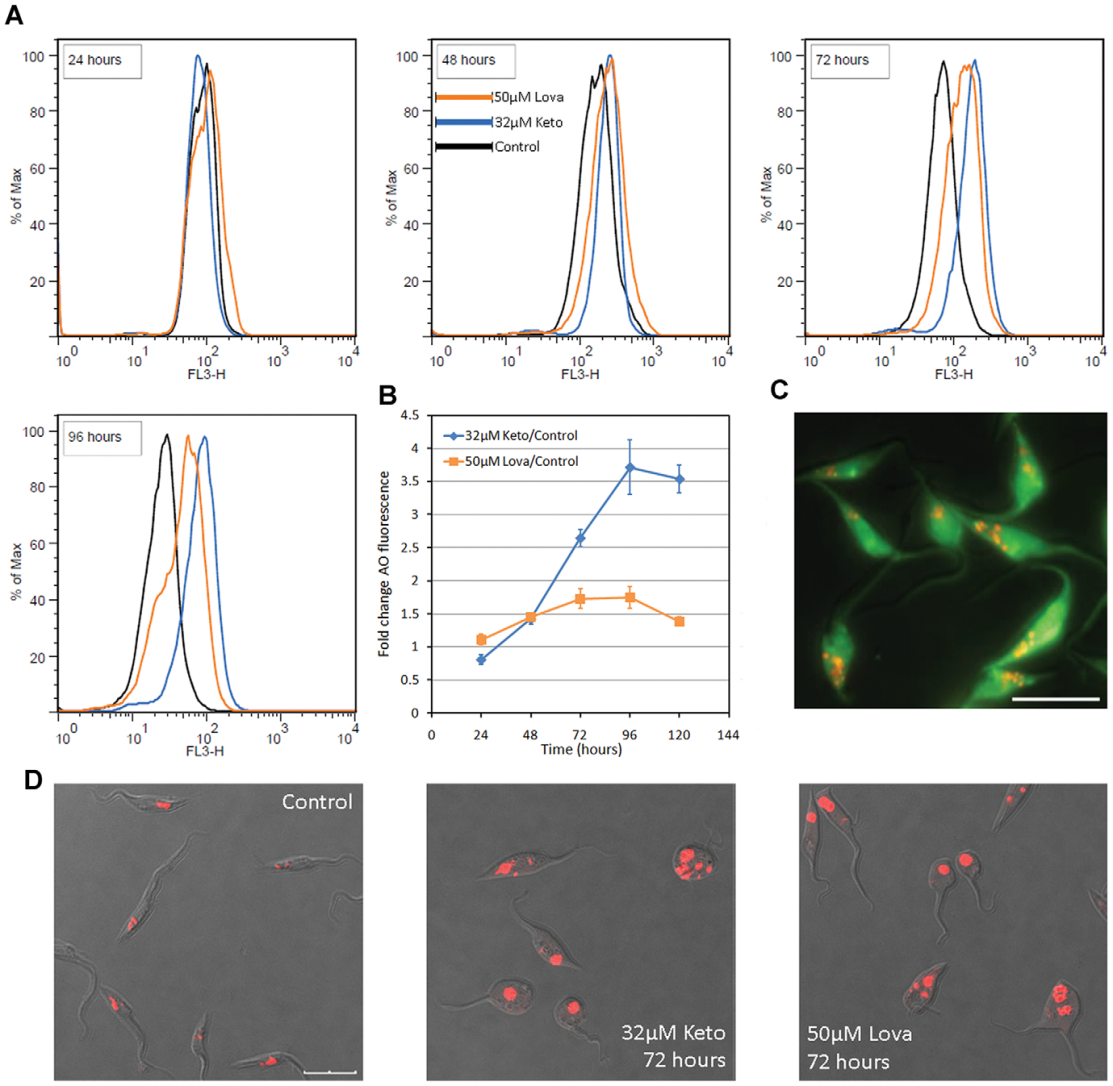
Swelling of the reservosomes in response to SBIs at the EC_50_/72 h, as shown by AO fluorescence. (A) Overlay flow cytometry histograms of SBI-treated cultures, for exposure times of 24 to 96 hours (in box). A gradual increase in red wavelength fluorescence (FL3-H) is observed in treated parasites. Histograms representative of triplicate experiments are shown. (B) Fold-change of the geometric mean of AO fluorescence intensity with respect to control cells in triplicate flow cytometry experiments. (C) Fluorescence microscopy of a control culture, showing acidic vesicles (reservosomes) stained in red with AO; the scale bar indicates 10 µm. (D) Confocal microscopy of live parasites, showing the increase in size of the acidic vesicles in the posterior region of treated cells. Overlay images of DIC and red fluorescence channels are shown and the scale bars indicate 10 µm.

For confirmation of this swelling of the reservosome in response to the SBIs, flow cytometry and fluorescence microscopy were performed with the acidotrophic dye AO, which accumulates in *T. cruzi* reservosomes [41] (Figure 3C). Flow cytometry analysis of parasites treated with SBIs at the EC_50_/72 h showed an increase in fluorescence intensity in the red light range after 48 hours of exposure (Figure 3A and B). This increase in fluorescence intensity was time-dependent, and was more evident in ketoconazole-treated cells. The visualization of living parasites stained with AO by confocal microscopy (Figure 3D), or of paraformaldehyde-fixed parasites stained with LysoTracker Red (Figure S2) also confirmed the increase in the size of acidic vesicles in the posterior region of the cells. AO can also stain acidocalcisomes [42], but the size and position of the stained organelles clearly indicated that they were reservosomes. Taken together, these data suggest that reservosome hypertrophy is a response to incubation with the ketoconazole or lovastatin at the EC_50_/72 h.

**Figure 4:**
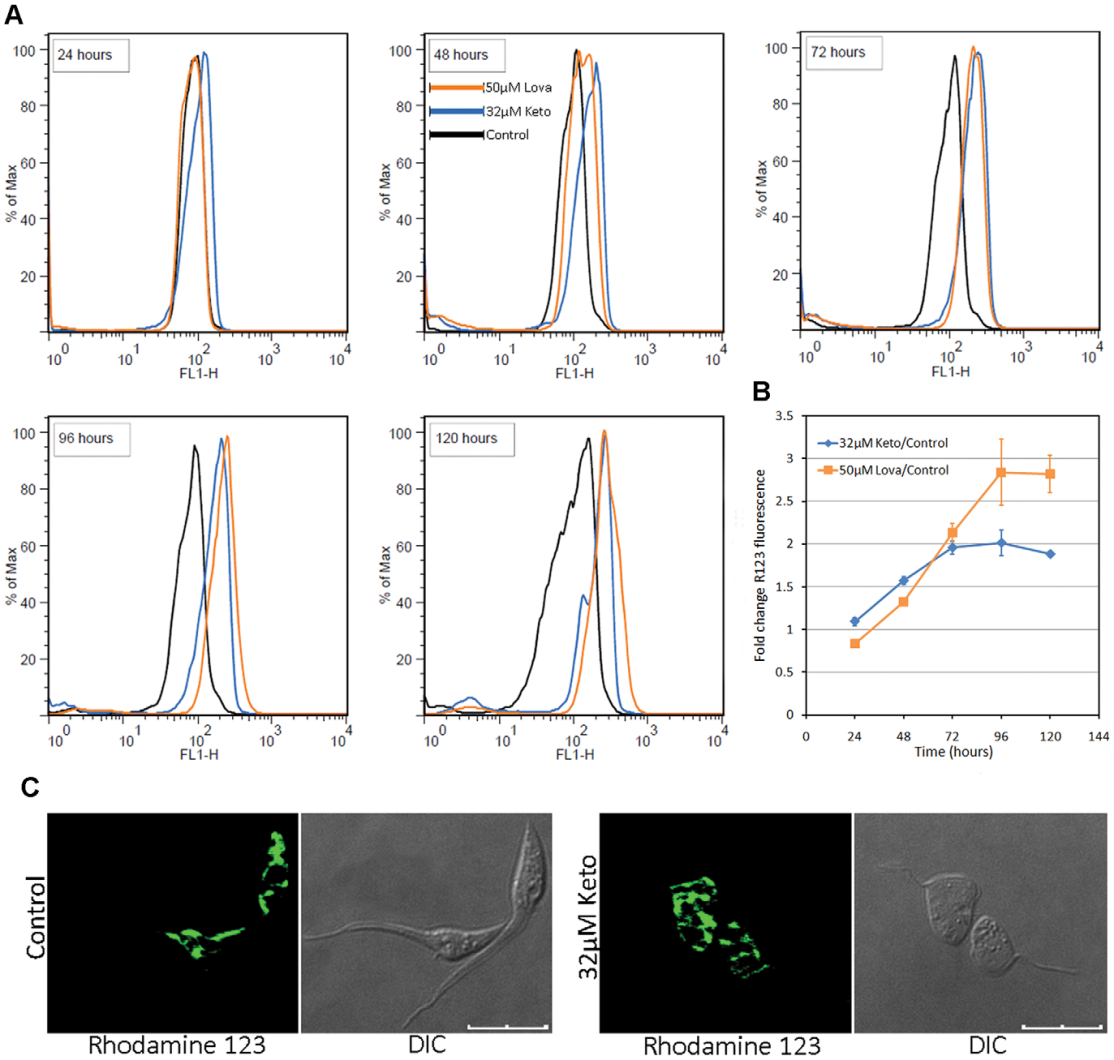
Mitochondrial branching in response to SBIs at the EC_50_/72 h, as detected by R123 fluorescence. (A) Overlay histograms of flow cytometry experiments on SBI-treated cultures, for exposure times of 24 to 120 hours (in box). A gradual increase in green wavelength fluorescence (FL1-H) can be seed for the treated parasites. Histograms representative of triplicate experiments are shown. (B) Fold-change in the geometric mean of R123 fluorescence intensity (FL1-H) with respect to control cells in triplicate flow cytometry experiments. (C) Confocal microscopy of live parasites, showing the branching of the mitochondrial membranes in treated parasites (72 hours); the results for lovastatin were similar to those for ketoconazole and are therefore not shown here; the scale bars indicate 10 µm.

Treatment with either of the SBIs at the EC_50_/72 h also resulted in mitochondrial disorganization, with abnormal branching of the mitochondrial membranes throughout the parasite body (Figure 2C, D, G and H). By contrast to the previously demonstrated swelling of the mitochondria in response to some SBIs [43], [44], [45], [46], [47], [48], we observed extensive branching of the mitochondrial membranes and their packing into trabecular structures (Figure 2C, G and H). In some instances, it was possible to observe concentric patterns of the inner mitochondrial membrane in contact with the kinetoplast (Figure 2D) and, at higher magnification, it was possible to see the pattern of the cristae (Figure 2C, in box). This mitochondrial remodeling was related to the presence of myelin figures (Figure 2G), suggesting the occurrence of mitophagy or the involvement of mitochondrial membranes in autophagosome assembly [49]. The proliferation of mitochondrial membranes was analyzed with R123, a cationic lipophilic fluorescent dye, the distribution of which depends on the inner membrane potential maintained by respiring mitochondria. R123 fluorescence intensity increased after 48 hours of exposure to SBIs (Figure 4A and B). This increase was not due to an increase in cell size, because the treated parasites maintained a normal pattern of light scattering (FSCxSSC) and displayed little change in shape over up to 96 hours of drug exposure (Figure S1). The visualization of R123-labeled live parasites by confocal microscopy confirmed the proliferation of mitochondrial membranes (Figure 4C). After 72 hours of drug exposure, R123 staining in treated cells was more intense for lovastatin than for ketoconazole (Figure 4B). This may reflect differences in the cellular distributions of the targets of these two SBIs. Together with the electron microscopy results, these data show that functional mitochondrial membranes proliferate in response to incubation with ketoconazole or lovastatin at the EC_50_/72 h.

Even after 5 days of treatment with SBIs at the EC_50_/72 h, we observed no phosphatidylserine exposure or internucleosomal DNA fragmentation (Figure S3) — classic characteristics of apoptotic cell death. Hence, the late cell death observed probably occurred by an exacerbated autophagic process, due to the absence of mature endogenous sterols.

### Rapid necrotic cell death after incubation with SBIs at the EC_100_/24 h

**Figure 5:**
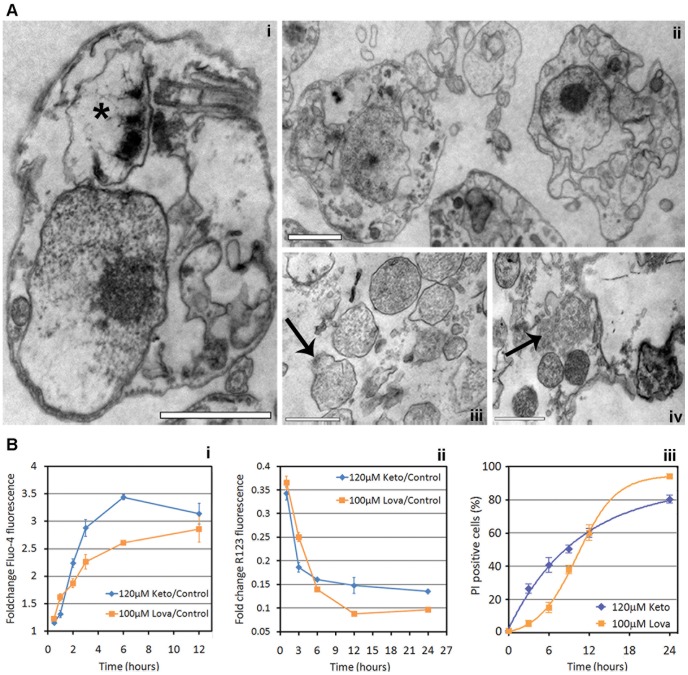
TEM and flow cytometry assays after treatment with SBIs at the EC_100_/24 h. (A) TEM images after 18 hours of exposure to SBIs at the EC_100_/24 h, showing marked cell degradation, with kinetoplast disruption (* in (i)) and cell lysis (ii); the occurrence of reservosome lysis is highlighted in (iii) and (iv). These morphological patterns are similar to those observed during cell death by necrosis. TEM results were similar for the two SBIs and the drug name is not shown. Scale bars: (i) and (ii), 1 µm; (iii) and (iv), 0.5 µm. (B) Flow cytometry analysis of *T. cruzi* necrotic death in reponse to SBIs at the EC_100_/24 h. (i) Analysis of relative intracellular calcium concentrations by fluo-4-AM staining, after 0.5 to 12 hours. A rapid increase in fluo-4-AM fluorescence with respect to control cells can be seen after exposure to the two SBIs at the EC_100_/24 h. (ii) Assay of mitochondrial membrane depolarization by R123 staining; time-dependent mitochondrial depolarization can clearly be seen by comparison with control cells. (iii) Cell viability analysis based on propidium iodide staining; the percentage dead cells (PI-positive) is plotted as a function of drug exposure time. Note the differences in cell lysis kinetics for the two drugs: the experimental points were optimally adjusted by a sigmoidal curve for lovastatin and by a negative exponential curve for ketoconazole. The raw flow cytometry plots can be seen in Figure S4.

Lethal doses of lovastatin and ketoconazole induced drastic morphological changes in the cells. After 12 hours of exposure to the SBI at the EC_100_/24 h, the parasites were spherical and displayed intense vacuolization, as seen on stained smears (Figure 1F). Ultrastructural analysis by TEM showed intense morphological changes, including an increase in cell volume, organelle swelling (Figure 5A-i and 5A-ii), reservosome lysis (Figure 5A-iii and 5A-iv) and a loss of plasma membrane integrity (cell lysis) (Figure 5A-ii). These morphological patterns are consistent with necrotic cell death [50], [51].

Plasma membrane rupture is a hallmark of necrotic cell death [51]. We further investigated this aspect by quantifying the internalization of the vital dye propidium iodide by flow cytometry. We observed time-dependent cell lysis for both SBIs, with more than 80% of cells lysed after 24 hours of exposure (Figure 5B-iii). The kinetics of cell lysis differed for the two drugs, following a sigmoidal curve for lovastatin and a negative exponential curve for ketoconazole, possibly reflecting differences in the toxic effects of these SBIs. Dead cells are smaller than living cells, so the percentage of cells that have been lysed can also be determined from the FSCxSSC pattern (Figure S4C), as recently demonstrated for cell death in *Trypanosoma brucei* [52].

An increase in cytosolic calcium concentration is known to be an essential initial event in cell death by necrosis [50]. Flow cytometry assays with the calcium fluorophore fluo-4-AM [53] indicated that cytoplasmic calcium concentration increased within one hour of exposure to the SBIs (Figure 5B-i). At short exposure times (30 to 60 minutes), cytosolic calcium influx was more intense in response to lovastatin than in response to ketoconazole (Figure 5B-i). As increases in cytosolic calcium concentration may result in mitochondrial overload and mitochondrial transmembrane depolarization [54], , we also used R123 staining and flow cytometry to quantify mitochondrial membrane potential. We observed strong, time-dependent mitochondrial depolarization for both SBIs. Considering only viable cells gated on the basis of FSCxSSC pattern, R123 fluorescence intensity in parasites exposed to drug treatment for one hour was only about one third that in control cells (Figure 5B-ii). These data suggest that mitochondrial depolarization is one of the first events in the response to incubation with ketoconazole or lovastatin at the EC_100_/24 h.

**Figure 6:**
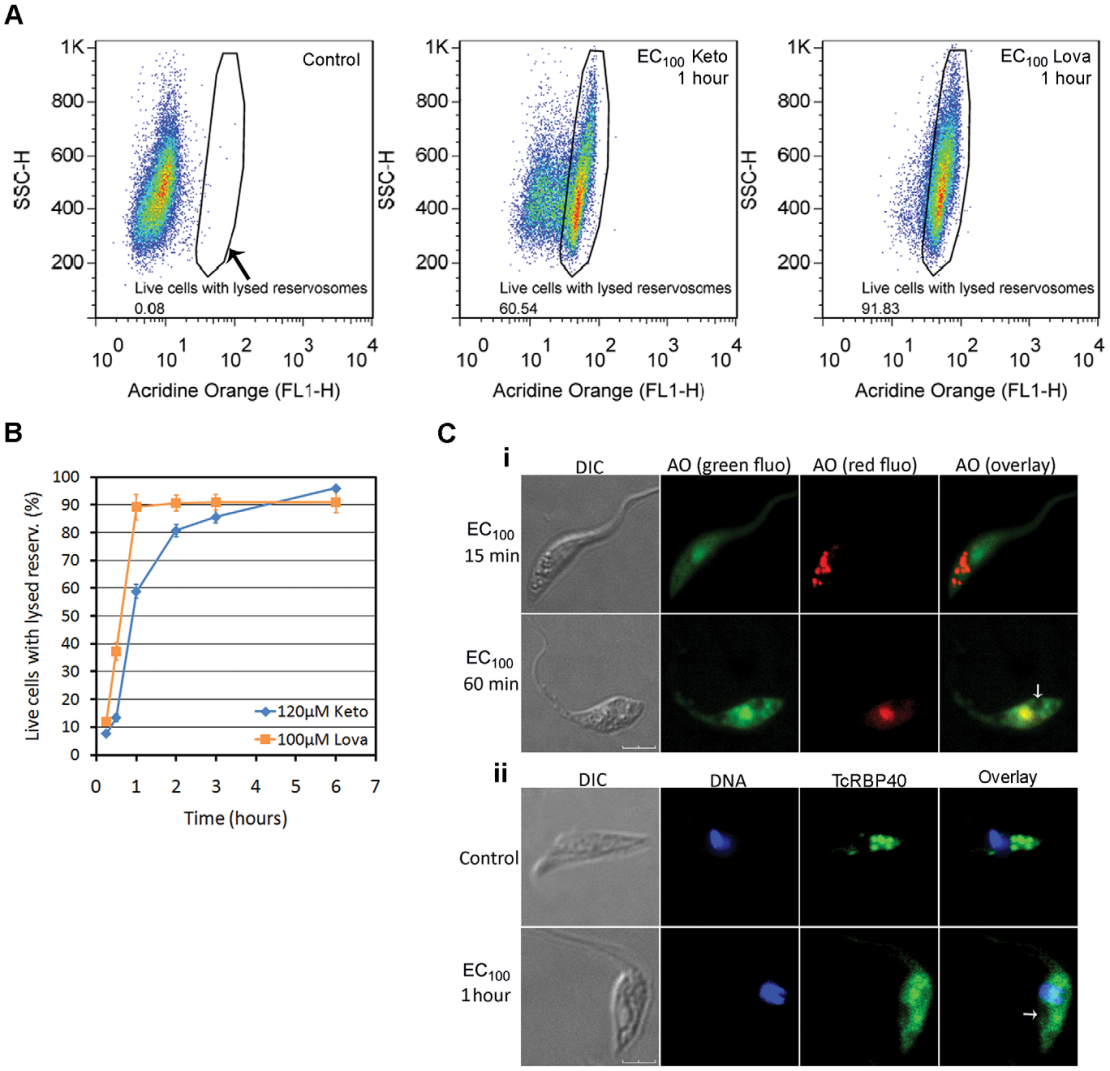
Reservosome membrane permeabilization (RMP) in response to treatment with SBIs at the EC_100_/24 h. (A) Example of RMP analysis by flow cytometry with AO, showing that treated cells with lysed reservosomes have a high FL1-H signal intensity. The values inside the boxes indicate the percentage of cells with lysed reservosomes. (B) Kinetics of RMP obtained by flow cytometry; each experimental point indicates the mean and standard deviation of triplicate experiments. (C) Visualization of reservosome lysis by confocal microscopy; (i) Live cells stained with AO; green (“green fluo”) and red (“red fluo”) AO fluorescence was photographed in different frames. The normal pattern of reservosome staining persists after 15 minutes of drug exposure (upper row), but, within 1 hour, all the acidic vesicles disappear (bottom row). (ii) Immunofluorescence analysis with an antibody directed against a reservosomal protein (TcRBP40); in control cells, this protein is found mostly in the reservosomes (upper row). However, after 1 hour of drug treatment, a diffuse signal is observed throughout the parasite body (bottom row). In (i) and (ii), the white arrows indicate low-fluorescence regions possibly corresponding to sites previously occupied by intact reservosomes. For both confocal experiments, similar results were obtained for ketoconazole and lovastatin and the drug used is therefore not indicated. The scale bars indicate 4 µm (i) and 3 µm (ii).

Our TEM data indicated that reservosome lysis occurred after incubation with the SBIs at the EC_100_/24 h (Figure 5A-iii and 5A-iv). We tested this hypothesis further, by incubating treated cells with AO and then analyzing reservosomes by fluorescence microscopy and flow cytometry. As recently shown for necrotic cell death in *Dictyostelium* [38], AO fluorescence at the green wavelength increased after lysosome rupture, due to the release of AO from lysosomes into the cytosol. Flow cytometry analysis of cells treated with SBIs at the EC_100_/24 h showed a time-dependent shift in FL1-H signal intensity, from pale green to bright green (Figure 6A). Reservosome membrane permeabilization (RMP) occurred rapidly, after as little as 15 minutes of drug exposure (Figure 6B). After short periods of exposure (30 to 120 minutes), the RMP was more intense for lovastatin than for ketoconazole, possibly reflecting more intense cytoplasmic calcium overload over short periods of exposure for lovastatin-treated cells (Figure 5B-i). Fluorescence confocal microscopy showed that most parasites retained a normal pattern of reservosome staining for the first 15 minutes of drug exposure (Figure 6C-i, upper row) but, the acidic vesicles disappeared within an hour of treatment (Figure 6C-i, bottom row), leaving a translucent posterior region at sites probably previously occupied by intact reservosomes (Figure 6C-i, white arrow). Reservosomes were lysed at about the time after which the recovery of cell growth was no longer possible following the withdrawal of the drug from the culture medium (compare Figure 1C and D with Figure 6B). This suggests the RMP is the “point of no return” in the necrotic cell death induced by high doses of SBIs.

We investigated RMP further, by immunofluorescence studies with an anti-serum against TcRBP40 protein, which is found preferentially in reservosomes in control cells [40] (Figure 6C-ii, upper row). Following treatment with the SBIs at the EC_100_/24 h, the parasites presented no TcRBP40 staining in the reservosome, a weak signal instead being found throughout the parasite (Figure 6C-ii, bottom row). These data suggest that soluble reservosome proteins, such as TcRBP40, are released into the cytoplasm after short periods of exposure to the SBIs at the EC_100_/24 h.

**Figure 7:**
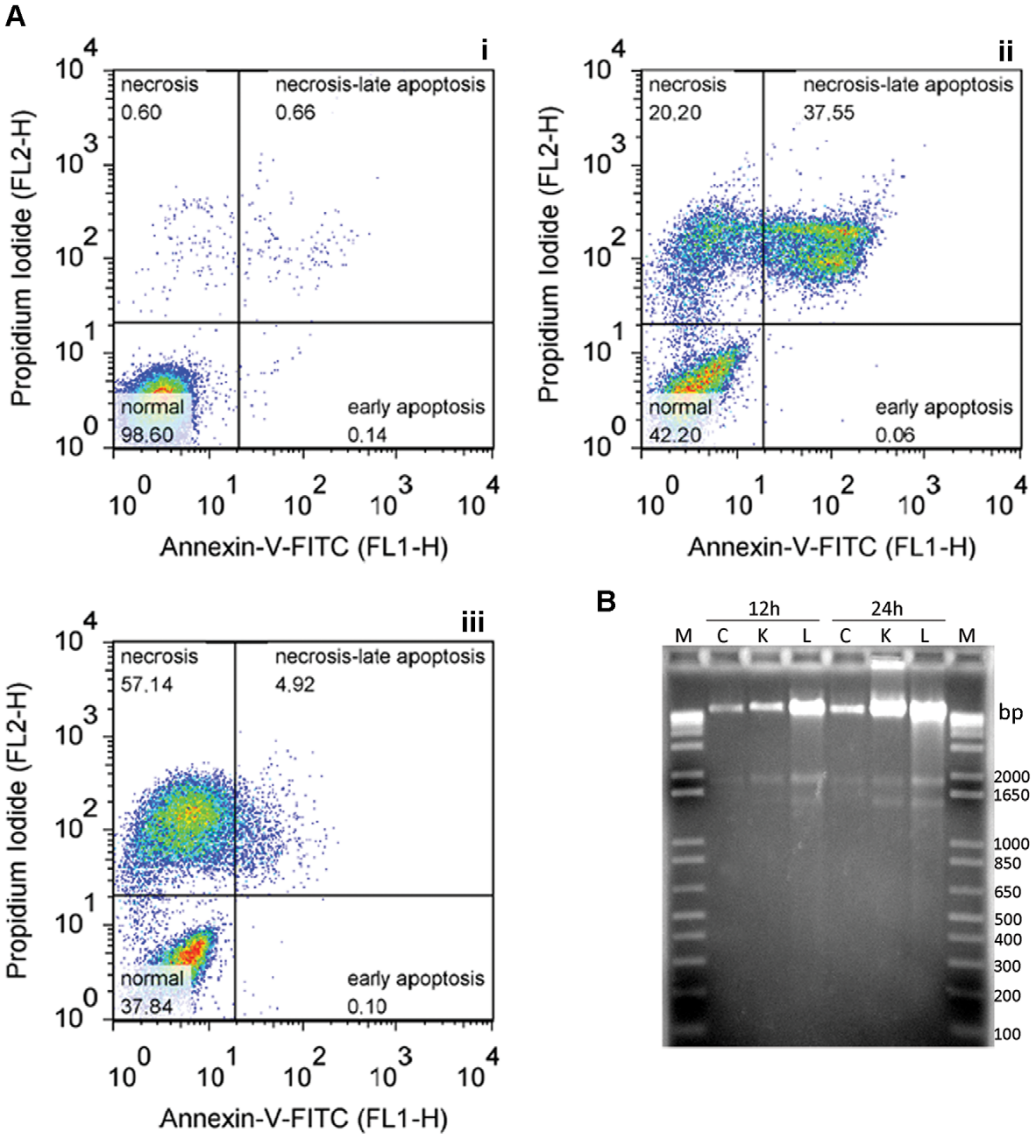
Absence of apoptotic markers in the EC_100_/24 h response. (A) Analysis of phosphatidylserine exposure by co-staining with Annexin-V-FITC and PI. As an example, we have plotted data for exposure for 12 hours to ketoconazole (ii) or lovastatin (iii), together with the control cell pattern (i). (B) DNA laddering assay; total DNA was isolated from control cultures (C) and from drug-treated cells (120 µM ketoconazole (K), 100 µM lovastatin (L)), after 12 or 24 hours of exposure (indicated at the top), as described in the methods section. We subjected 5 µg of the DNA to electrophoresis in a 1.5% agarose gel stained with ethidium bromide; M lanes contain the 1 kb Plus DNA ladder.

Apoptosis can again be excluded, due to the absence of phosphatidylserine exposure and internucleosomal DNA fragmentation (Figure 7). Furthermore, *in situ* TUNEL assays (Figure S5) showed that, even at later exposure times (12 and 24 hours), few cells displayed nuclear DNA fragmentation but most cells displayed kinetoplast labeling, indicating mitochondrial DNA fragmentation, consistent with electron microscopy observations (Figure 5A-i). Taken together, these results indicate that the EC_100_/24 h of SBIs induced necrotic death in *T. cruzi*.

## Discussion

We show here that the incubation of *T. cruzi* epimastigotes with the SBIs ketoconazole and lovastatin triggers different types of cell death at different doses. At the EC_50_/72 h, SBIs caused delayed cell death by autophagy, after about six days of exposure, whereas, at higher doses (EC_100_/24 h) SBI treatment led to rapid necrotic death within hours. These differences highlight the importance of studying programmed cell death (PCD) in unicellular organisms, such as trypanosomatids, which is a relatively new idea [56]. Our results also suggest that the cell death pathway activated depends on the type and intensity of the stimulus [57].

Several studies have shown that the inhibition of endogenous sterol biosynthesis by SBIs results principally in changes to the composition and function of membranes [58], [59], causing major morphological changes in *T. cruzi* and *L. major*, mostly affecting the single tubular mitochondrion of these parasites [11], [60]. The incubation of *T. cruzi* with inhibitors of C14-DMT [45], [46], sterol methyltransferase [43], [48] and squalene synthase [44] results in disorganization of the mitochondrial membrane, followed by intense swelling and loss of the contents of the matrix. Such changes were also observed in response to ketoconazole and lovastatin in this study. However, in addition to the previously reported swelling of the mitochondria, we also observed previously unreported changes to the mitochondria in response to SBIs: intense proliferation of the inner mitochondrial membrane, which appeared to be highly branched and compact. Our analysis with R123 indicated that functional proliferation of the inner mitochondrial membrane had occurred, because this dye stains only biochemically active mitochondria [61]. Inhibitors of sterol methyl-transferase have recently been shown to alter the function of the *L. amazonenzis* mitochondrion, preventing it from generating or sustaining the H^+^ electrochemical gradient driven by respiration [62]. Our confocal microscopy observations indicated a weaker, punctate R123 signal along the mitochondrial membrane, suggesting that the H^+^ electrochemical gradient was weaker in drug-treated parasites. The greater R123 fluorescence intensity in treated parasites on flow cytometry may therefore be due to the branching of the inner mitochondrial membrane, resulting in higher levels of R123 accumulation within cells [63]. The observed circularization of mitochondrial cristae may result from the disorganization and subsequent fusion of inner membranes in the absence of mature endogenous sterols [60]. In this context, the presence of myelin figures in close contact with mitochondria may indicate the degradation of damaged mitochondrial membranes by mitophagy and/or the involvement of mitochondrial membranes in autophagosome assembly, as recently demonstrated in starvation-induced autophagy [49]. The possible involvement of mitochondria in *T. cruzi* autophagosome biogenesis requires further investigation. We are currently producing *T. cruzi* cell lines expressing fluorescent proteins tagged to the autophagosome marker ATG-8 as a tool for addressing this issue.

By contrast, given the mitochondrial location of the mevalonate pathway enzymes HMG-CoA synthase and HMG-CoA reductase in *T. cruzi* ([64], reviewed in [11]) and the presence of endogenous sterols in the inner membrane of this organelle [65], mitochondrial inner membrane branching in response to SBIs at the EC_50_/72 h may be a direct response to the depletion of endogenous sterols, highlighting the importance of this organelle in the ergosterol biosynthesis of trypanosomatids. After longer periods of drug exposure, higher levels of branching were observed with lovastatin than with ketoconazole, possibly because the target of lovastatin (HMGR) is located in the mitochondrion [64], whereas that of ketoconazole (C14-DMT) is located in the endoplasmic reticulum and reservosomes [66].

Another morphological change observed in response to treatment with ketoconazole or lovastatin at the EC_50_/72 h was an increase in reservosome size. Reservosomes are found exclusively in the *Schizotrypanum* subgenus, in which they take the form of spherical organelles concentrated in the posterior region of *T. cruzi* epimastigotes; they are thought to be prelysosomal compartments in which material from endocytosis accumulates [67]. No typical lysosomes have ever been found in *T. cruzi* (reviewed in [68]), so the reservosomes were recently given the name “lysosome-related organelles” (LRO), due to their acidic pH (∼6) and the presence of acidic hydrolases (cruzipain and serine carboxypeptidase) ([69], reviewed in [41]). The progressive hypertrophy of this organelle in the face of sterol inhibition was recently demonstrated following treatment with ketoconazole [47]. We show here that reservosome size also increases in response to lovastatin. A recent analysis of reservosome content by mass spectrometry showed the presence of two enzymes responsible for the final steps in ergosterol biosynthesis (sterol 24-C-methyltransferase and sterol C-24 reductase) [70]. Together with the recent demonstration of the presence of C14-DMT in this organelle [66], it seems that the increase in reservosome size following SBI treatment reflects the involvement of this organelle in sterol biosynthesis. As for mitochondrial branching, the more intense swelling of the reservosome observed after treatment with ketoconazole than after treatment with lovastatin may reflect differences in the cellular distributions of the targets of these drugs. However, the abnormal increase in reservosome size may also be due to the autolysomal function of this organelle, as the material engulfed by autophagsomes is delivered to this organelle for degradation during autophagy [71]. In this context, reservosome hypertrophy and mitochondrial branching may be signals of intense autophagy.

As reservosomes accumulate material acquired by endocytosis [37], their increase in size may reflect an increase in endocytic activity due to the depletion of endogenous sterols. This hypothesis is supported by the increase in exogenous cholesterol concentration observed in *Leishmania* treated with the same classes of inhibitors used here (azoles and statins) [72]. Thus, the abnormally large reservosomes observed may reflect both changes in sterol biosynthesis and autolysosome functions, by also increases in endocytosis.

These findings, together with those of other studies, indicate that the depletion of endogenous sterols by SBIs and the consequent accumulation of abnormal lipids induces the formation of autophagic vacuoles and myelin figures in the cytoplasm [43], [44], [45], [47], both these features being characteristic of autophagic cell death [51]. An *in silico* analysis of trypanosomatid genomes demonstrated the presence of the core proteins required for autophagy (Atg3, 4, 7, and 8) [71], [73], with the Atg8 conjugation system working in a similar manner to its homologs in yeast and humans [71]. Even after five days of exposure to the SBIs at the EC_50_/72 h, the parasites displayed no phosphatidylserine exposure or internucleosomal DNA fragmentation, probably reflecting an absence of apoptosis in *T. cruzi* after SBI treatment. The late cell lysis observed after several days of drug exposure probably therefore involves a type of secondary necrosis [74] after uncontrolled autophagy. These data contrast with those for mammalian cells suggesting that apoptotic death occurs in response to lovastatin [75], [76], [77], [78], [79] and ketoconazole [80], [81], at doses similar to the EC_50_/72 h determined here.

By contrast to what was observed in the EC_50_/72 h experiments, the treatment of *T. cruzi* with high doses of SBIs (EC_100_/24 h) resulted in rapid cell death. This death probably occurred via a toxic mechanism independent of endogenous sterol levels [28], but this experimental model is nonetheless an interesting tool for studying mechanisms of programmed cell death in *T. cruzi*. There are several forms of PCD. Necrotic cell death (NCD) was long considered to be an uncontrolled or accidental form of cell death occurring in response to intense physicochemical stress (such as mechanical force or high temperature) and lacking the features of apoptosis or autophagy. However, there is growing evidence to suggest that NCD may be a mechanism governed by a set of signal transduction pathways and catabolic mechanisms [50], [82]. NCD involves an increase in cell volume (oncosis), the swelling of organelles (particularly mitochondria), an absence of chromatin condensation and disruption of the plasma membrane, leading to a loss of intracellular components [50], [51]. The intracellular events specific to NCD occur in the following order: failure of calcium homeostasis leading to the accumulation of calcium ions in the cytoplasm, early mitochondrial dysfunction (including ATP depletion and the generation of reactive oxygen species), perinuclear clustering of organelles, activation of proteases (mostly calpains and cathepsins), permeabilization/lysis of lysosomes and cell lysis [50]. These events do not, individually, define NCD, but their accumulation in an organized cascade provides strong evidence of this process [50], [51].

The response of *T. cruzi* to SBIs at the EC_100_/24 h, despite the non physiological nature of this physicochemical stress, induced almost all the features of NCD listed above, including the morphological changes and biochemical events. Previous studies have shown that the single mitochondrion of this parasite can accumulate large amounts of calcium, inducing permeabilization of the inner mitochondrial membrane, with a major impact on the electron transport chain, resulting in mitochondrial oxidative damage followed by cell death [55]. It has recently been shown that the treatment of *T. cruzi* with cramoll 1,4, a seed lectin isolated from *Cratylia mollis*, induces an increase in cytoplasmic calcium concentration accompanied by the accumulation of calcium ions in the mitochondria, followed by an increase in the production of reactive oxygen species (ROS), a decrease in mitochondrial membrane potential and an absence of oxidative phosphorylation, leading to NCD with no DNA fragmentation [54]. Given the rapid accumulation of calcium ions in the cytoplasm, the concomitant mitochondrial depolarization and the absence of DNA fragmentation observed here, the NCD observed in response to high doses of SBIs probably involves the accumulation of calcium ions in the mitochondrion, leading to the generation of ROS. These are the initial molecular steps leading to RMP and time-dependent cell lysis, the hallmarks of necrotic cell death. Furthermore, as EGTA did not interfere with cytoplasmic calcium overload (Figure S4A), this ion must arise from intracellular pools, probably in the endoplasmic reticulum and/or acidocalcisomes [83].

Recent studies of NCD in *Dictyostelium* have shown that mitochondrial uncoupling and ROS production are early events, occurring about 20 minutes after the induction of death and triggering the cascade of events involved in NCD [84]. Mitochondrial changes can usually be reversed by removing the death-inducing factor [85], [86]. By contrast, lysosomal membrane permeabilization, which occurs after 70 to 100 minutes in *Dictyostelium* [38], is a “point of no return” event culminating in cell lysis after about 150 minutes of NCD activation. Thus, the correlation between RMP kinetics and commitment to cell death indicates that RMP represents the “point of no return” event in *T. cruzi* NCD. The extensive cellular degradation observed by microscopy is probably triggered by the release of reservosomal proteases. Recent TEM studies have described reservosome rupture in response to trypanocidal drugs [47], [87], [88], but this is the first demonstration of the importance of RMP during *T. cruzi* cell death by complementary methods (TEM, flow cytometry and confocal microscopy). It is not yet possible, from the results presented, to identify the intermediate steps leading to RMP, but the activation of a calpain-cathepsin cascade triggered by cytoplasmic calcium [89], [90], [91] and/or direct oxidative damage [92], [93] may be crucial.

The *T. cruzi* development stages residing in the mammalian host (amastigotes and bloodstream trypomastigotes) are the main targets of SBI treatment. Typical reservosomes storing material from endocytosis are visible only in epimastigote forms of *T. cruzi*, but all developmental stages present lysosome-related organelles [69] and permeabilization of the reservosome (lysosome) membrane may play a crucial role in controlling cell death in mammalian stages of the parasite too. However, as amastigotes are 10 times more sensitive to SBIs than other stages [12], additional pathways may also contribute to cell death in these cells. The next step in our initial cellular and molecular characterization of the response of *T. cruzi* to SBIs will therefore involve the performance of these assays on amastigotes. Furthermore, given the limited therapeutic utility of the drug analyzed here, we will also test other SBI in future studies.

**Figure 8:**
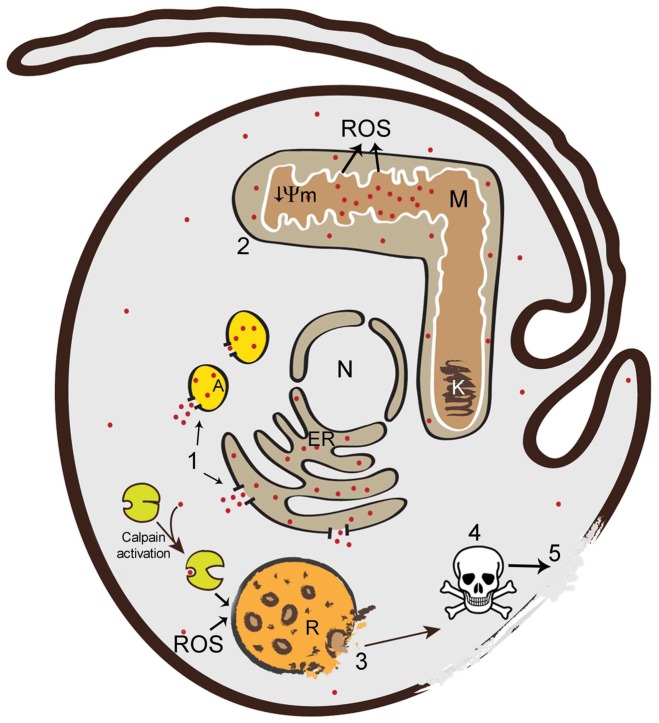
Model of *T. cruzi* necrotic cell death. The cellular events during the necrotic death of epimastigotes were reconstructed from the results of this and published studies. The events occur in the following order: 1: cytoplasmic calcium overload from acidocalcisomes and/or the ER (red dots represent Ca^2+^); 2: accumulation of Ca^2+^ in the mitochondria, leading to inner membrane depolarization (↓Ψm) and ROS (reactive oxygen species) production; 3: RMP, due to the action of ROS and/or Ca^2+^- activated calpains, potentially corresponding to the point of no return in the necrotic pathway; 4: extensive cell degradation by proteases released from the reservosomes; 5: cell lysis. N: nucleus, ER: endoplasmic reticulum, M: mitochondria, K: kinetoplast, R: reservosome; A: acidocalcisome.

Nevertheless, using classical SBIs acting on the epimastigote stage, we were able to obtain new insight into the response of *T. cruzi* to ergosterol synthesis inhibition. Based on the results of this work and those of published studies, we propose a model of *T. cruzi* necrotic cell death (Figure 8). The stress caused by the drugs first induces a rapid cytoplasmic calcium overload (Figure 8, event 1). The mitochondria concomitantly accumulate large amounts of calcium, impairing electron transport and leading to mitochondrial oxidative damage and inner membrane depolarization [54] (Figure 8, event 2). The ROS generated by mitochondria [54] and/or calcium-activated cytoplasmic calpains then act directly on the reservosome membrane, inducing RMP, the “point of no return” in the necrotic pathway (Figure 8, event 3). The leakage of reservosomal proteases into the cytoplasm leads to high levels of cell degradation (Figure 8, event 4) and time-dependent cell lysis (Figure 8, event 5), hallmarks of necrotic cell death. The results for both the EC_50_/72 h and the EC_100_/24 h provided no evidence of phophatidylserine exposure or nuclear DNA fragmentation at these doses in *T. cruzi*, excluding the occurrence of classical apoptotic PCD. By contrast, a recent study with human osteosarcoma cells showed that even high concentrations of ketoconazole (100–200 µM) induced an apoptotic PCD mediated by caspase 3 that culminated in nuclear DNA fragmentation [81]. Despite the weak inhibition of human C14-DMT by ketoconazole [94] and other possible mechanisms of action in human cells [95], these results demonstrate that the same drug can activate different death pathways in *T. cruzi* and human cells. This may reflect the absence of classical caspases in trypanosomatids, in the genomes of which only distant orthologs have been identified, encoding metacaspases. The role of *T. cruzi* metacaspases is unknown, but recent findings suggest they may have essential functions in cell death regulation, cell cycle progression and differentiation [35], [96].

The occurrence of autophagic and necrotic cell death processes with characteristics similar to those in other cell models, in an ancient protozoan parasite, provides support for the notion of conserved mechanisms of cell death in eukaryotes [97]. The lack of apoptosis in the response of *T. cruzi* to ketoconazole and lovastatin, by contrast to what has been reported for mammalian cells treated with the same drugs, at concentrations similar to those used here, points to a recent emergence of caspase-dependent apoptosis in the evolution of cell death. The identification of events conserved in distant eukaryotes, such as mammals and protozoa, is essential for an understanding and the identification of possible functional modules, molecules and mechanisms specific to each type of cell death. Furthermore, the induction of PCD with drugs could potentially be exploited in the development of new trypanocidal drugs.

## Supporting Information

Figure S1 **Morphological changes in response to treatment with SBIs at the EC_50_/72 h, as observed by light microscopy analysis of stained smears.** Left column: control cells; middle column: ketoconazole; right column: lovastatin. The numbers on the left side of the figure refer to the number of days of drug exposure. Black arrows indicate cells with two nuclei and/or kinetoplast. The highlighted boxes show details of cells with large numbers of acidic vesicles in their posterior parts. Bars indicate 10 µm, except for highlighted cells (0.5 µm).
(TIF)

Figure S2 ***T. cruzi*** **staining with the acidotophic fluorescent dye LysoTracker® Red (LTR) DND-99 (Invitrogen).** The parasites were treated for 72 hours with 32 µM ketoconazole (row 2), 50 µM lovastatin (row 3) or left untreated (row 1), stained with 0.5 µM LTR and fixed for fluorescence microscopy analysis. Column A: DIC; column B: DNA dye Hoechst 33342; column C: acidic vesicles stained with LTR; column D: superimposition of B and C. Note the stronger staining in the posterior region of SBI-treated parasites.
(TIF)

Figure S3 **Absence of apoptotic markers in the EC_50_/72 h response.** (A) Analysis of phosphatidylserine exposure, based on double-staining with annexin-V-FITC and PI. As an example, data are plotted for 120 hours of exposure to 32 µM ketoconazole (ii) or 50 µM lovastatin (iii), and the control cell pattern is shown (i). (B) DNA laddering assay; total DNA was isolated from control cultures (0) and from drug-treated cells after 24 to 120 hours of drug exposure (indicated at the top). We separated 5 µg of DNA by electrophoresis in a 1.5% agarose gel andstained with ethidium bromide; M lanes contain the 1 kb Plus DNA ladder. Similar results were indicated for the two SBIs and the name of the drug used is therefore not indicated.
(TIF)

Figure S4 **Flow cytometry analysis of** ***T. cruzi*** **necrotic death in response to treatment with SBIs at the EC_100_/24 h of SBIs.** (A) Overlay histograms of Fluo-4-AM-stained cultures (with or without 1 mM EGTA) exposed to 100 µM lovastatin (i) or 120 µM ketoconazole (ii) from 0.5 to 12 hours. (B) Mitochondrial membrane depolarization; plots in (i) and (ii) show overlay histograms of R123-stained cultures exposed to EC_100_/24 h of lovastatin and ketoconazole, respectively; time-dependent mitochondrial depolarization with respect to control cells is clearly visible (iii). (C) Cell viability analysis; the percentage dead cells was determined by staining with the vital dye propidium iodide (ii) or from light scatter pattern (i) (data for 12 hours of exposure to lovastatin are plotted as an example).
(TIF)

Figure S5 **Analysis of DNA fragmentation in an** ***in situ*** **TUNEL assay.** Parasites were treated for 12 hours with the EC_100_ dose of ketoconazole or lovastatin (indicated on the left) and fixed for TUNEL experiments. DNase I-treated parasites were used as a positive control for the assay, together with normal parasites (negative control). DNA was stained with Hoechst 33342, and images were artificially colored in green to improve the visualization of overlay images. Note the TUNEL staining mostly in the kinetoplast region of SBI-treated parasites. Bars indicate 10 µm.
(TIF)
