# Supplementary figures and images for: Trypanosoma cruzi Response to Sterol Biosynthesis Inhibitors: Morphophysiological Alterations Leading to Cell Death
Source: PLoS One. 2013 Jan 31;8(1):e55497. doi: 10.1371/journal.pone.0055497 (PMC3561218; doi:10.1371/journal.pone.0055497)

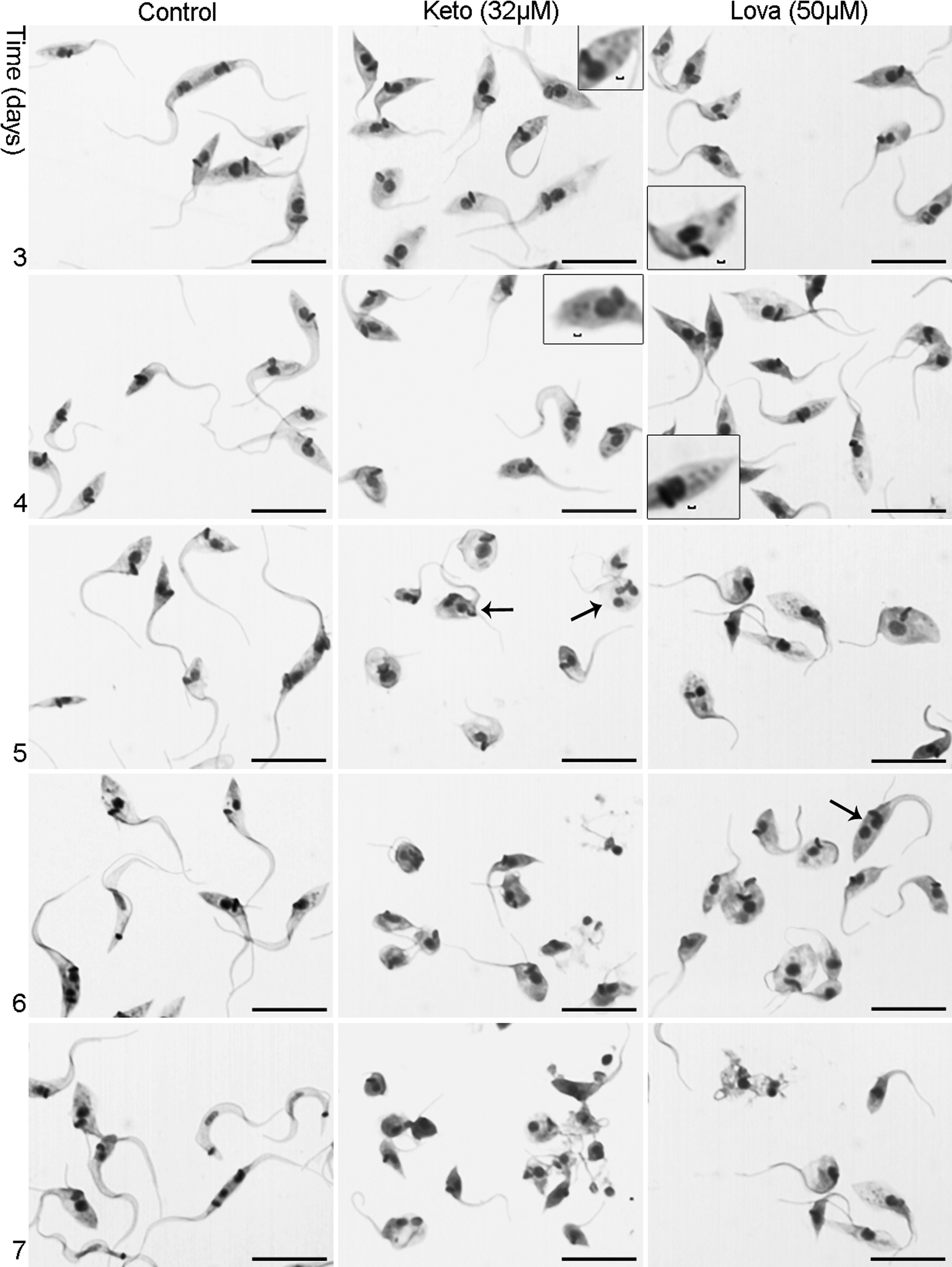

Supplement: Figure S1 — Morphological changes in response to treatment with SBIs at the EC50/72 h, as observed by light microscopy analysis of stained smears. Left column: control cells; middle column: ketoconazole; right column: lovastatin. The numbers on the left side of the figure refer to the number of days of drug exposure. Black arrows indicate cells with two nuclei and/or kinetoplast. The highlighted boxes show details of cells with large numbers of acidic vesicles in their posterior parts. Bars indicate 10 µm, except for highlighted cells (0.5 µm). (TIF) [file pone.0055497.s001.tif]

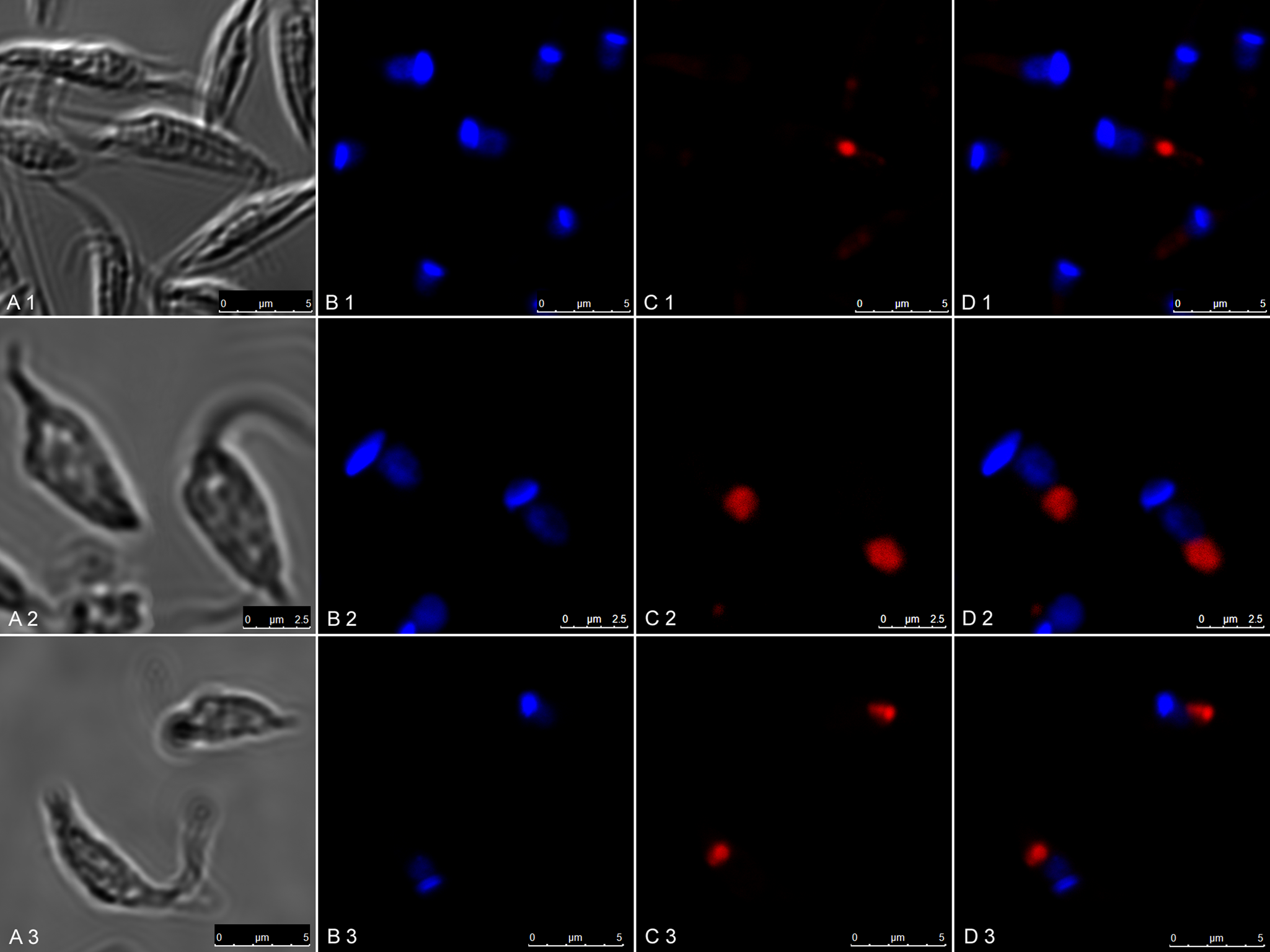

Supplement: Figure S2 — T. cruzi staining with the acidotophic fluorescent dye LysoTracker® Red (LTR) DND-99 (Invitrogen). The parasites were treated for 72 hours with 32 µM ketoconazole (row 2), 50 µM lovastatin (row 3) or left untreated (row 1), stained with 0.5 µM LTR and fixed for fluorescence microscopy analysis. Column A: DIC; column B: DNA dye Hoechst 33342; column C: acidic vesicles stained with LTR; column D: superimposition of B and C. Note the stronger staining in the posterior region of SBI-treated parasites. (TIF) [file pone.0055497.s002.tif]

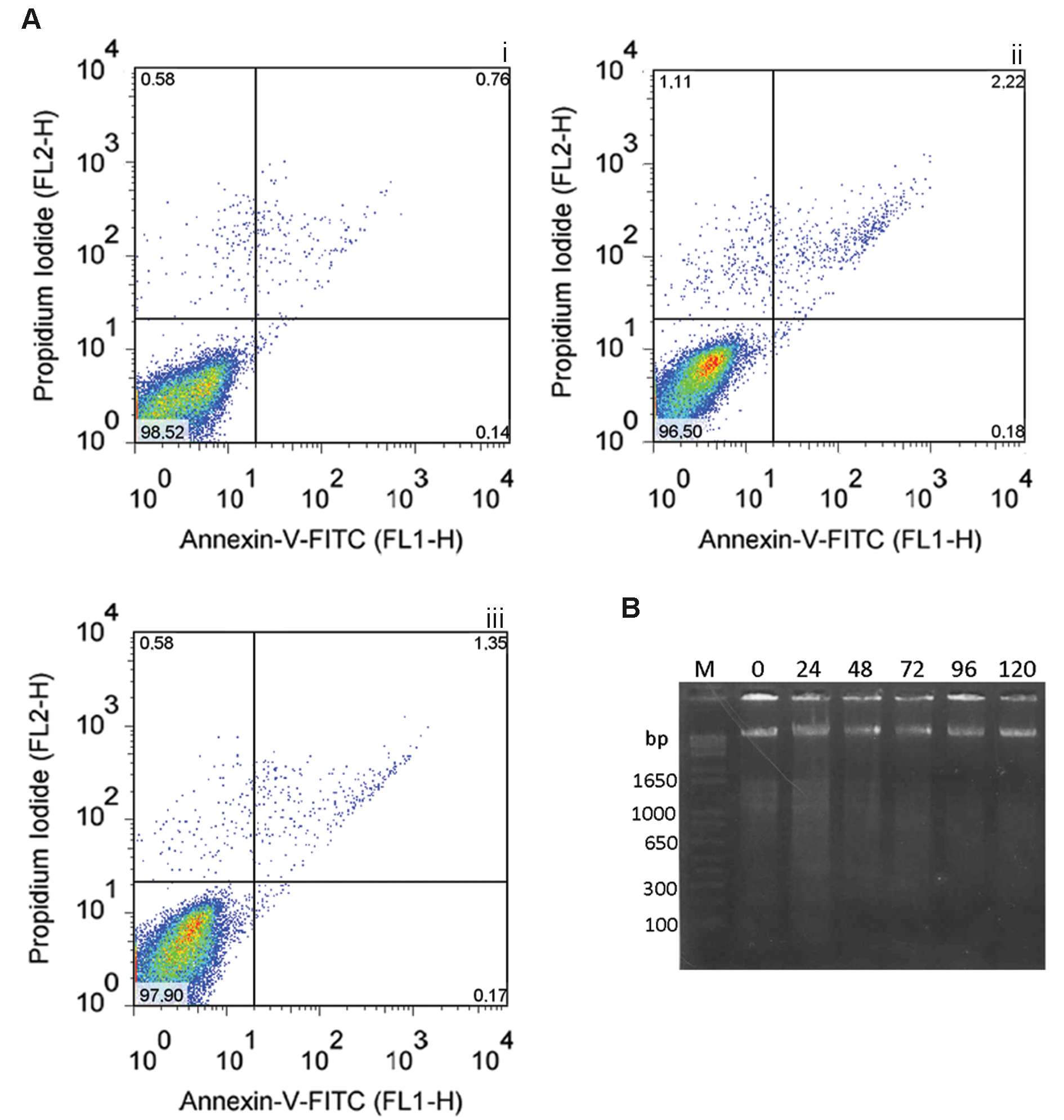

Supplement: Figure S3 — Absence of apoptotic markers in the EC50/72 h response. (A) Analysis of phosphatidylserine exposure, based on double-staining with annexin-V-FITC and PI. As an example, data are plotted for 120 hours of exposure to 32 µM ketoconazole (ii) or 50 µM lovastatin (iii), and the control cell pattern is shown (i). (B) DNA laddering assay; total DNA was isolated from control cultures (0) and from drug-treated cells after 24 to 120 hours of drug exposure (indicated at the top). We separated 5 µg of DNA by electrophoresis in a 1.5% agarose gel andstained with ethidium bromide; M lanes contain the 1 kb Plus DNA ladder. Similar results were indicated for the two SBIs and the name of the drug used is therefore not indicated. (TIF) [file pone.0055497.s003.tif]

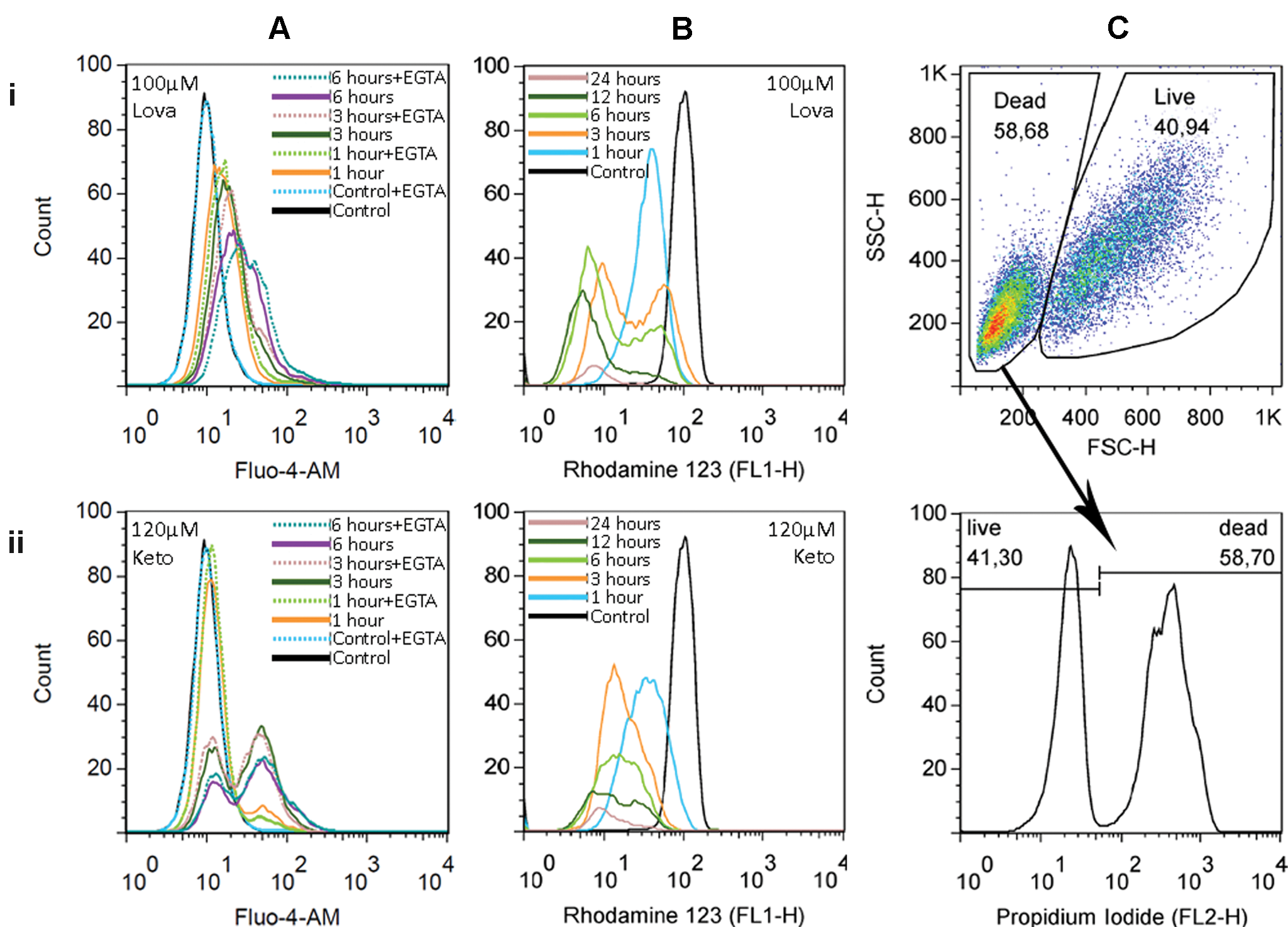

Supplement: Figure S4 — Flow cytometry analysis of T. cruzi necrotic death in response to treatment with SBIs at the EC100/24 h of SBIs. (A) Overlay histograms of Fluo-4-AM-stained cultures (with or without 1 mM EGTA) exposed to 100 µM lovastatin (i) or 120 µM ketoconazole (ii) from 0.5 to 12 hours. (B) Mitochondrial membrane depolarization; plots in (i) and (ii) show overlay histograms of R123-stained cultures exposed to EC100/24 h of lovastatin and ketoconazole, respectively; time-dependent mitochondrial depolarization with respect to control cells is clearly visible (iii). (C) Cell viability analysis; the percentage dead cells was determined by staining with the vital dye propidium iodide (ii) or from light scatter pattern (i) (data for 12 hours of exposure to lovastatin are plotted as an example). (TIF) [file pone.0055497.s004.tif]

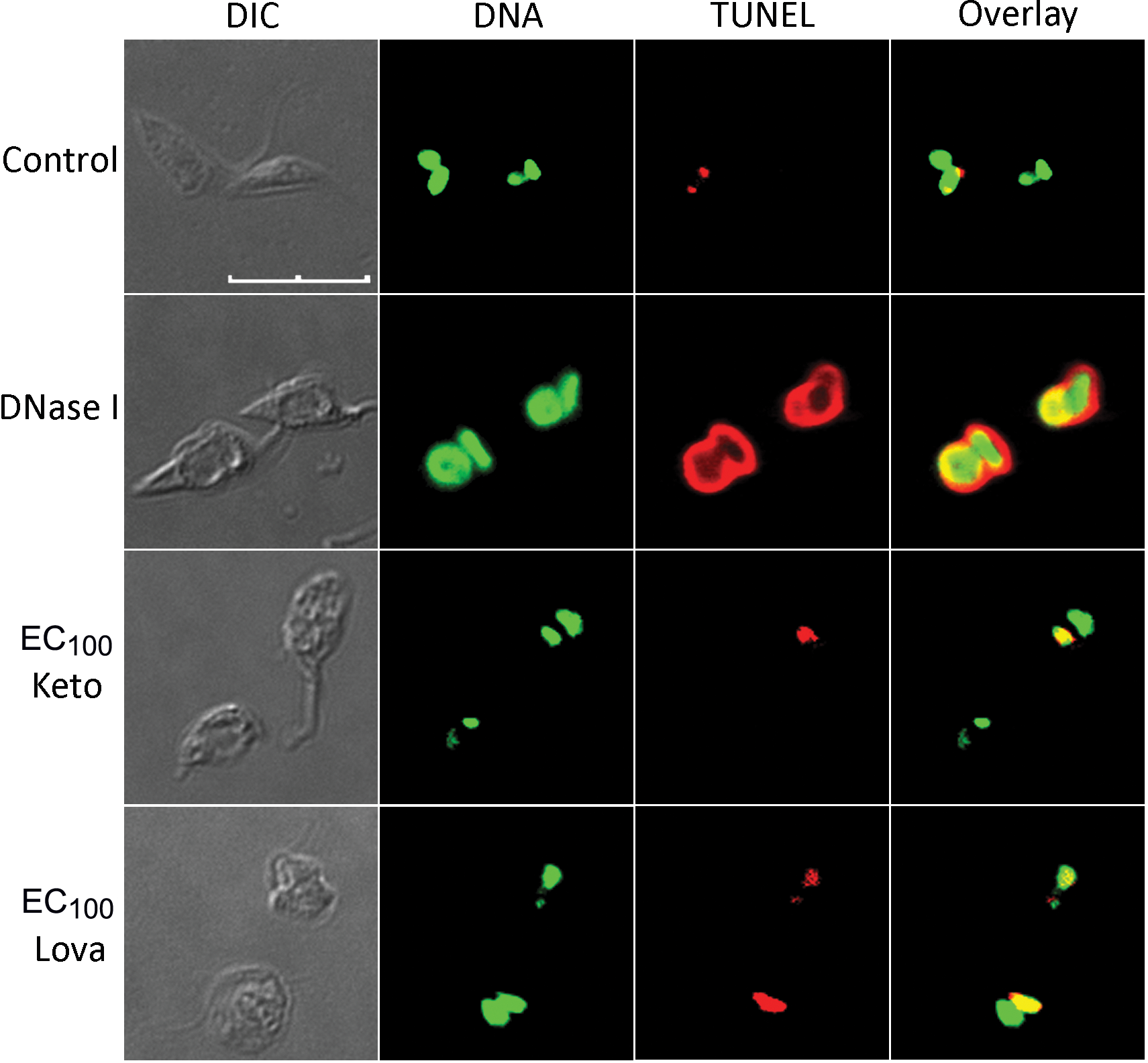

Supplement: Figure S5 — Analysis of DNA fragmentation in an in situ TUNEL assay. Parasites were treated for 12 hours with the EC100 dose of ketoconazole or lovastatin (indicated on the left) and fixed for TUNEL experiments. DNase I-treated parasites were used as a positive control for the assay, together with normal parasites (negative control). DNA was stained with Hoechst 33342, and images were artificially colored in green to improve the visualization of overlay images. Note the TUNEL staining mostly in the kinetoplast region of SBI-treated parasites. Bars indicate 10 µm. (TIF) [file pone.0055497.s005.tif]
